# No Evidence of the “Weekend Effect” in the Northern New South Wales Telestroke Network

**DOI:** 10.3389/fneur.2020.00130

**Published:** 2020-02-26

**Authors:** Thomas Lillicrap, Alex Pinheiro, Ferdinand Miteff, Pablo Garcia-Bermejo, Shyam Gangadharan, Thomas Wellings, Billy O'Brien, James Evans, Khaled Alanati, Andrew Bivard, Mark Parsons, Christopher Levi, Carlos Garcia-Esperon, Neil Spratt

**Affiliations:** ^1^Department of Neurology, John Hunter Hospital, Newcastle, NSW, Australia; ^2^School of Medicine and Public Health, University of Newcastle, Newcastle, NSW, Australia; ^3^Department of Neurology, Gosford Hospital, Gosford, NSW, Australia; ^4^Department of Neurology, Royal Melbourne Hospital, Melbourne, VIC, Australia; ^5^SPHERE, Sydney, NSW, Australia

**Keywords:** telestroke, weekend effect, thrombolysis, thrombectomy, door to needle time

## Abstract

**Background:** Admission outside normal business hours has been associated with prolonged door-to-treatment times and poorer patient outcomes, the so called “weekend effect. ” This is the *first* examination of the weekend effect in a telestroke service that uses multi-modal computed tomography.

**Aims:** To examine differences in workflow and triage between in-hours and out-of-hours calls to a telestroke service.

**Methods:** All patients assessed using the Northern New South Wales (N-NSW) telestroke service from April 2013 to January 2019 were eligible for inclusion (674 in total; 539 with complete data). The primary outcomes measured were differences between in-hours and out-of-hours in door-to-call-to-decision-to-needle times, differences in the proportion of patients confirmed to have strokes or of patients selected for reperfusion therapies or patients with a modified Rankin Score (mRS ≤ 2) at 90 days.

**Results:** There were no significant differences between in-hours and out-of-hours in any of the measured times, nor in the proportions of patients confirmed to have strokes (67.6 and 69.6%, respectively, *p* = 0.93); selected for reperfusion therapies (22.7 and 22.6%, respectively, *p* = 0.56); or independent at 3 months (34.8 and 33.6%, respectively, *p* = 0.770). There were significant differences in times between individual hospitals, and patient presentation more than 4.5 h after symptom onset was associated with slower times (21 minute delay in door-to-call, *p* = 0.002 and 22 min delay in door-to-image, *p* = 0.001).

**Conclusions:** The weekend effect is not evident in the Northern NSW telestroke network experience, though this study did identify some opportunities for improvement in the delivery of acute stroke therapies.

## Introduction

The John Hunter and Gosford Hospitals provide telestroke services to five rural hospitals within the Hunter New England Local Health District and the adjacent Mid-North Coast Local Health District (New South Wales, Australia), together representing ~1.1 million inhabitants in an area over 143,000 km^2^. John Hunter Hospital functions as the hub of the network, with Gosford Hospital providing Neurologist consultations via the network but not accepting patient transfers. The telestroke network aims to identify patient candidates for acute reperfusion therapies; thrombolysis, which is delivered at the spoke hospital and mechanical thrombectomy (MT) which requires transfer to John Hunter Hospital (1). Both therapies are time-sensitive and require developed acute stroke pathways to minimize door-to-thrombolysis (needle) (DTN) and door to groin puncture times (2–4).

After-hours presentation has been associated with prolonged DTN time and poorer patient outcomes (5–7), the so-called “weekend effect.” Studies in the USA (8) and Germany (9) have indicated that telestroke services are less prone to the weekend effect, but this has not been examined telestroke services that offer mechanical thrombectomy (MT) nor any telestroke service that relies on multi-modal computed tomography (mCT) imaging for treatment decisions. Furthermore, previous studies in telestroke networks have been confined to patients who received thrombolysis, and did not examine differences in the accuracy of triage by spoke-site staff, which has implications for telestroke resource management. In this study, we aimed to determine whether admission in- or out-of business hours had a significant influence on door to needle timings and accuracy of emergency department triage or patient outcomes in our telestroke network. In secondary analyses we examined differences between individual hospitals, differences between groups of hospitals sorted by experience in the telestroke network and the effects of factors such as stroke severity and time from symptom onset to presentation on hospital timings. The latter was examined both before and after the criteria for reperfusion therapy was extended to 24-h.

## Methods

### Ethics Approval

Ethics approval was gained from the Hunter New England Human Research Ethics Committee (HNEHREC Reference No: 13/02/20/5.06) with a posterior amendment (AU201712-15). Given the nature of the study (an audit of internal data) the requirement for individual patient consent was waived.

### Northern NSW Telestroke Network

The telestroke network commenced with the first spoke hub (hospital A) in April 2013, followed by Hospital B (2014), and three other sites (hospitals C, D, and E) joined in 2017. Of these hospitals, only Hospital B has neurologists on site and routinely provides in-hours thrombolysis while the telestroke network primarily functions out-of-hours. The telestroke network offers 24/7 services to the rest of the hospitals, with Neurologist workforce being drawn from both John Hunter Hospital and Gosford District Hospital, while John Hunter Hospital also accepts patient transfers for MT. The distance from spoke to hub ranges from 167 to 423 km. As part of the network, the rural hospitals were equipped with telemedicine cameras, the physicians were trained in acute stroke triage, and multimodal CT (compendium of brain non-contrast computed tomography -NCCT-, CT angiography -CTA-, and CT perfusion -CTP-) was introduced and performed routinely. Patients presenting with neurological symptoms as defined by the FAST scale (10) within the relevant time-frame from symptom onset would receive multimodal CT imaging and be assessed via the telestroke service. From April 2013 to November 2017, the stroke call criteria required symptom onset within 4.5 h. From November 2017, after publication of the DAWN (2) and DEFUSE-3 (11) trials, the time window for stroke calls was expanded to 24 h. Acute imaging interpretation and final treatment decision are performed by the consulting telestroke Neurologist, with spoke-hospital staff delivering thrombolysis and/or arranging transfer to John Hunter hospital for MT as appropriate. In cases where the patient received both thrombolysis and MT, thrombolysis would be initiated at the spoke site prior to transfer. For more details about our telestroke workflow, we direct the reader to our previous results (1, 12).

### Data Collection

All patients assessed using the Northern New South Wales (NSW) telestroke service from April 2013 to January 2019 were eligible for inclusion and were only excluded from the analysis if complete data were not available. Clinical data were retrospectively collected from April 2013 to June 2016 (46 patients, 8.5% of the total) and prospectively collected June 2016–January 2019. In addition to baseline demographics, past medical history, baseline National Institutes of Health Stroke Scale (NIHSS), treatment decision, final diagnosis, and several time points were collected. The DTN time was defined as the time between emergency department (ED) presentation and delivery of the bolus of thrombolytics. DTN was divided into three parts; the time between arrival at the ED and call to the telestroke neurologist (“door-to-call”), the time from the call to the decision (reperfusion treatment, yes or no) (“call-to-decision”), and the time from this decision to thrombolysis, where applicable (“decision-to-needle”). Door-to-image, defined as the time between arrival at ED to first image of brain CT was also collected. Patients who suffered a stroke in hospital were excluded from door-to-call and door-to-imaging analyses but were included in all others. The time between symptom onset (either witnessed onset or time last known well) and ED presentation was analyzed as <3 h, 3–4.5 h, and >4.5 h as 4.5 h is the currently licensed window for thrombolysis.

Final diagnosis was coded as stroke [confirmed ischemic lesion on multimodal CT or follow-up CT or magnetic resonance image (MRI)] or not confirmed stroke. Transient ischaemic attacks (TIAs) were not classed as confirmed stroke unless the transient perfusion deficit was visible on acute CTP and consistent with clinical presentation. Symptomatic intracranial hemorrhage was defined according to the Safe Implementation of Treatments in Stroke Monitoring Study (SITS-MOST) criteria (13). Telestroke calls were classified as “In-hours” (i.e., business hours) if the call was made between 8:00 a.m. and 5:00 p.m. local time Monday-Friday, excluding New South Wales public holidays. The rest of the calls were considered as “out-of-hours.”

### Statistical Analysis

All statistical analysis was performed on Stata version 14 (Statacorp, USA). Differences in triage between in-hours and out-of-hours were assessed by comparing the proportion of patients in each category who were confirmed to have stroke, and the proportion who received acute reperfusion therapy using Pearson's chi-squared test. Timings of the stroke workflow in the hub sites were assessed using linear regression. To examine the effect of hospital experience in the telestroke network, the three hospitals that have joined it most recently (hospitals C, D and E) were grouped together as “least experienced” while Hospital A was coded as “most experienced” Hospital B, with an intermediate level of experience was excluded from the analysis. Another regression analysis was performed with the patients since the time-window for acute therapies was expanded to 24 h to examine the effects of symptom onset to presentation time after this change in call criteria.

## Results

A total of 674 patients were assessed from April 2013 to January 2019; 135 were excluded due to incomplete data leaving 539 (80%) patients in this analysis. Of these, 256 (47.5%) were assessed in-hours and 283 (52.5%) out-of-hours; 20 calls related to patients already in hospital. One hundred and twenty-two patients (23.3%) underwent reperfusion therapies (75 thrombolysis, 25 thrombectomy, and 22 combined thrombolysis and thrombectomy) (Table 1). A total of two patients were treated who were not found to have confirmed stroke. After final work-up, one of these patients was still believed to be a stroke, while the other was diagnosed with an unspecified mimic. A total of two patients suffered symptomatic intracerebral hemorrhage (sICH) after reperfusion therapy (1.6% of those treated).

**Table 1:** Patient population characteristics in-hours vs. out-of hours and treated vs. non-treated with acute reperfusion therapy.

	**In-hours patients**	**Out-of-hours patients**	**Patients receiving acute reperfusion therapy**	**Patients not receiving acute reperfusion therapy**	**All patients**
Total number of patients (%, male)	256 (58%)	283 (59%)	122 (62%)	401 (57%)	539 (59%)
Age (Mean, range)	69 (29–96)	70 (23–95)	69 (29–92)	70 (23–96)	70 (23–96)
NIHSS (Median–IQR)	4 (2–8)	5 (2–10)	10 (6–17)	3 (1–7)	4 (2–9)

Triage efficacy was similar in-hours to out-of-hours, with 71.5% of patients confirmed to have stroke and 22.7% selected for reperfusion therapies in hours compared to 73.9% confirmed strokes and 22.3% selected for reperfusion therapy out of hours (*p* = 0.560 and 0.930, respectively) (Table 2). Patient outcomes were also similar between the two groups (34.7% 90-days mRS ≤ 2 in-hours vs. 33.6% out-of-hours, *p* = 0.770). Forty-six patients were thrombolysed in hours, with 51 thrombolysed out-of-hours. The median door-to-needle time was 91 min (IQR 71–110 min), being longer out-of-hours than in-hours (94 vs. 87 min, respectively). This pattern was the same for the decision to needle time (23 vs. 20 min) and the door to image time (56 vs. 53 min) although the call to decision time was slightly shorter out-of-hours (36.5 vs. 40.5 min). None of these differences were statistically significant after adjusting for other factors (hospital, onset-presentation time, stroke severity).

**Table 2:** Patients confirmed to have suffered a stroke and treated with reperfusion therapy (thrombolysis or MT), in- vs. out-of-hours.

	**In-hours *N* (% of in-hours calls)**	**Out-of-hours *N* (% of out-of-hours calls)**	**Total *N* (% of all calls)**
Total number of patients	256	283	539
Confirmed stroke	171 (67.6%)	197 (69.6%)	370 (68.6%)
Treated with reperfusion therapy	58 (22.7%)	64 (22.6%)	122 (22.6%)
90-day mRS ≤ 2	89 (34.8%)	95 (33.6%)	184 (34.1%)

### Effect of in-hours vs. Out-of-Hours

Patient arrival in-hours was not a statistically significant predictor of work-flow times, despite a trend for it to be associated with reduced times such as an estimated 10.2-minute decrease in door-to-call times (95% CI −1.8 to 22.2, *p* = 0.097), and an estimated 6.1-min (95% CI −6.16 to 18.35, *p* = 0.329) decrease in door-to-image and 4.33 min (95% CI −1.00 to 4.33, *p* = 0.109) decrease in decision-to-needle times compared to out-of-hours presentation. None of these differences were statistically significant (all *p* > 0.05); including the overall door to needle time (estimated 9.6 min, 95% CI −28.9 to 9.8, *p* = 0.330).

### Onset to Presentation Time

A total of 296 patients presented within 3 h of onset, 31 between 3 and 4.5 h and 169 patients presented more than 4.5-h since the last time seen well. There was no significant difference in any of the acute stroke metrics (door-to-image, door-to-call, or decision-to-needle times) between patients admitted within 3 and 3–4.5 h after onset (all nominal *p* > 0.05; see Table 3). The presentation over 4.5 h was associated with significantly slower door-to-call and door-to-image times though not with a slower decision-to-needle time (see Table 3). There was a trend for a slower call-to-decision time (by an estimated 5.2 min, 95% CI −0.6 to 11.1, *p* = 0.080).

**Table 3:** Estimated effects on workflow times (from linear regression) of symptom-onset to presentation time for all patients.

**Time from onset-ED**		**3–4.5 h**	**> 4.5 h**
Door-to-call time	β (95% CI)	2.6	21
		(−22.1 to 27.2)	(7.8 to 34.1)
	*p*	0.838	0.002
Call-to-decision time	β (95% CI)	−8.1 (−19.1 to 3.0)	5.24 (−0.6 to 11.1)
	*p*	0.15	0.08
Decision-to-needle time	β (95% CI)	−5.2 (−19.9 to 9.5)	2.7 (−6.8 to 12.1)
	*p*	0.485	0.573
Door-to-Image time	β (95% CI)	6.39 (−18.8 to 31.6)	22.1 (8.7 to 65.5)
	*p*	0.618	0.001

*All estimates are relative to patients presenting within 3 h of symptom onset*.

A specific analysis after expanding the call window to 24 h since onset was performed. This group comprised 407 patients in total, 63 treated with thrombolysis alone, 24 with MT alone and 19 with both therapies. Amongst this group, those presenting >4.5-h after onset still experienced significantly longer door-to-call, door-to-imaging and call-to-decision times (see Table 4); while those presenting 3–4.5 h after onset experienced a call-to-decision time faster than patients presenting within 3 h (by an estimated 14.5 min, 95% CI 1.5 to 27.5 min, *p* = 0.029).

**Table 4:** Estimated effects on workflow times (from linear regression) of symptom-onset to presentation time after the protocol was changed to include patients presenting up to 24-h after onset.

**Time from onset-ED**		**3–4.5 h**	**>4.5 h**
Door-to-call time	β (95% CI)	−13.8 (−43.4 to 14.8)	27.4 (13.0 to 41.7)
	*p*	0.343	<0.001
Call-to-decision time	β (95% CI)	−14.5	6.9 (0.4 to 13.4)
		(−27.5 to −1.5)	
	*p*	0.029	0.038
Decision-to-needle time	β (95% CI)	−0.4 (−18.5 to 17.8)	−1.4 (−13.1 to 10.2)
	*p*	0.967	0.809
Door-to-Image time	β (95% CI)	−13.7 (−42.8 to 15.4)	29.1 (14.5 to 43.6)
	*p*	0.356	<0.001

*All estimates are relative to patients presenting within 3 h of symptom onset*.

### Hospital Experience

Compared to Hospital A (the most experienced site), all sites except hospital C demonstrated significantly slower decision-to-needle times by ~10–12 min (see Table 5). Hospital B demonstrated slower workflow across all of the times while hospital D demonstrated significantly faster door-to-imaging times than any other site (Table 5). Hospital experience (that is, admission to the most experienced site relative to the 3 least-experienced sites) was associated with significantly faster decision-to-needle times (by an estimated 14.3 min, 95%CI 5.8 to 22.8 min, *p* = 0.001) but not faster door-to-call, call-to-decision or door-to-image times (*p*-values of 0.217, 0.819, and 0.861, respectively, see Supplementary Material for further details).

**Table 5:** Estimated differences in work-flow times relative to Hospital A after adjusting for other variables (*P < 0.05).

		**Door-to-call time**	**Call-to-decision time**	**Decision-to-needle time**	**Door-to-imaging time**
Hospital B	β	25.8 (6.9–44.7)	13.4	10.2 (2.2–18.3)	33.4
	(95% CI)		(5.0–21.8)		(14.1–52.8)
	*P*	0.008*	0.002*	0.014*	0.001*
Hospital C	β	2.9 (−25.6 to 1.3)	2.3	6.1 (−9.0 to 21.2)	0.2
	(95% CI)		(−10.4 to 15.1)		(−28.9 to 29.3)
	*P*	0.844	0.719	0.424	0.99
Hospital D	β	−1.6 (−18.7 to 15.5)	−0.3	12.5 (4.5 to 20.4)	−17.8
	(95% CI)		(−7.9 to 7.4)		(−35.1 to −0.4)
	*P*	0.856	0.94	0.003*	0.045*
Hospital E	β	17.0 (0.2 to 33.9)	1.3	10.2 (3.5 to 17.0)	18.8
	(95% CI)		(−6.2 to 8.8)		(1.6 to 35.9)
	*P*	0.048*	0.733	0.003*	0.032*

### Stroke Severity and Reperfusion Therapy

There was a trend for faster door-to-call times with more severe strokes, by ~1.7 min for every unit increase in NIHSS, but this was not statistically significance once other factors such as the use of reperfusion therapy were adjusted for (*p* = 0.081). Patients who did receive reperfusion therapy tended to have more severe strokes than those who did not (see Table 1) and were assessed more quickly on average. The use of reperfusion therapy (either thrombolysis or MT) was consistently associated with faster door-to-call (by 16.8 min, 95% CI 0.6 to 32.9 min, *p* = 0.043) and door-to-imaging (by 25.3 min, 95% CI 8.8 to 41.8 min, *p* = 0.003) times but was not significantly associated with the call-to-decision time (estimate = 0.7 min faster, *p* = 0.577).

## Discussion

There were no differences in the accuracy or speed of triage for the telestroke service between in-hours and out-of-hours. The proportion of patients confirmed to have stroke, the proportion who received reperfusion therapy and the proportion who were independent at 90 days were all similar between both groups. The lack of variation in mRS outcomes between the two groups is consistent with previous studies (8, 9); differences in the rates of stroke diagnosis and reperfusion therapy have not previously been examined in a telestroke network. Differences in acute stroke metrics were not statistically significant between in- and out-of-hours, although there were differences between hospitals. These results are reasonably consistent with the single study that has examined door-to-needle times in a telestroke network (8), which found a small difference (1.6 min) in one of their workflow times (page-camera, for which our study has no correlate) but no difference in overall door-needle times. Both telestroke analyses contrast with previous studies in comprehensive stroke centers that did find a significant difference between in-hours and out-of-hours, both in Australia (14) and overseas (7). The hypothesized reasons for these differences have included extra time required for the stroke team to travel to the hospital after being called or having a smaller and/or less experienced staff working out-of-hours. Our telestroke service does not require the stroke team to travel to the hospital, eliminating this potential source of delay. With regard to staffing levels, the spoke hospitals in the Northern NSW telestroke network tend to have lean staffing levels at all times, thus the difference in available staff between in-hours and out-of-hours is minimal.

Previous studies on the weekend effect in telestroke networks have been limited to date; studies in the USA (8) and Germany (9) have examined differences in patient outcomes between in-hours and out-of-hours presentations, and the former examined differences in workflow-timings (finding no difference in door-to-needle times between the two groups). However, both of these studies were confined to patients who received thrombolysis. There are no studies we are aware of that examine the weekend effect in a telestroke network that includes MT as a treatment option, and hence triages patients presenting outside the typical thrombolysis window. Furthermore, no previous study has examined a telestroke service that uses CTP routinely (both the US and German telestroke networks primarily relied on NCCT and CTA), nor have differences in the accuracy of patient triage for the telestroke service been examined previously. The latter are potentially important for managing the telestroke workload.

The routine use of CTP may, in theory, have differential effects outside of normal business hours due to the availability (or lack thereof) of radiography staff trained in this procedure. The use of perfusion imaging (either CTP or MR-Perfusion) is important for the selection of patients for MT in the extended time-window of 6- to 24-h after symptom onset, into which a significant proportion of our patients fall once the lengthy transfer times between spokes and hub are accounted for. Our group has recently published a comprehensive analysis of the effects of mCT on clinical decision-making and outcomes in this telestroke network (12). Amongst 80 patients who met standard NCCT and clinical criteria for thrombolysis, 36 (45%) were not thrombolysed based on mCT criteria, 6 because mCT demonstrated a large established core (>70 mL) that was not visible on NCCT, while the remainder had very small or no lesions and 12 of these patients were subsequently diagnosed as stroke mimics. In keeping with our previous larger observational study (15), clinical outcomes in patients with no or very small lesions were excellent without treatment.

The delays associated with patients presenting more than 4.5 h after stroke onset probably indicate an incomplete awareness of the expanded window for stroke therapy despite specific training being performed. The delay in triaging patients admitted out of the classic thrombolysis window is likely to reduce the efficacy of reperfusion therapy when it is provided and hence degrade patient outcomes (16). This will need to be a key focus of future training within the telestroke network.

Patients who received reperfusion therapies (thrombolysis or MT) were triaged faster, resulting in door-to-call and door-to-imaging times ~15 and 25 min, respectively, faster than patients who did not go on to receive such therapies. This suggests that staff are acting with more urgency when they judge that a patient may be suitable for reperfusion therapy early in the triage process. This may in part be due to greater stroke severity amongst patients who received reperfusion therapy, a hypothesis supported by the fact that stroke severity was an important confounder for the regression models without being statistically significant once reperfusion therapy had been corrected for.

The variation in timing between hospitals was considerable, and may account for an important proportion of the apparent difference between in-hours and out-of-hours timings given that hospital B, which demonstrated the slowest times across the board, also had the highest proportion of out-of-hours telestroke calls (66% out-of-hours vs. 53% across all sites, see Supplementary Table 1). Hospital B has local neurologists and primarily uses the telestroke service out-of-hours, or when the local Neurologists are unavailable. This may explain the slower timings at this hospital as well, since the local emergency staff works with two different models, leading to possible delays activating the telestroke team.

The effect of hospital experience was chiefly evident in differences in the decision-to-needle time. The spoke hospitals in the Northern NSW telestroke network tend to have relatively transient medical workforce, with a more stable nursing staff hence the accumulation of experience within a hospital primarily occurs within the nursing staff. This may be why the benefit of this experience is primarily demonstrated in the decision-to-needle time, which is influenced more by the efficiency of nursing staff than by the efficiency of the treating doctor.

The key strength of this study is the examination of a range of quality-of-care measures in addition to the standard patient outcomes, including the speed of work-flow for both patients who received reperfusion therapy and those who did not, the latter group having previously been excluded from examinations of the weekend effect in telestroke networks. The key weaknesses of the study lie in the fact that some of the data were collected retrospectively, and a considerable portion of patients were excluded due to incomplete data.

## Conclusions

This study has demonstrated that presentation out-of-hours is not a barrier to effective and efficient use of the telestroke service, and has identified opportunities for improvement within the Northern NSW telestroke network, in particular with regard to patients admitted more than 4.5 h after symptom onset.

## Data Availability Statement

The datasets generated for this study will not be made publicly available. The full data-set contains potentially identifiable patient data. Requests to access the dataset can be made to the corresponding author.

## Ethics Statement

The studies involving human participants were reviewed and approved by Hunter New England Health Human Research Ethics Committee. Written informed consent for participation was not required for this study in accordance with the national legislation and the institutional requirements.

## Author Contributions

In addition to these tasks, FM, PG-B, SG, TW, BO'B, JE, KA, CG-E, and NS collected and collated data for the article. TL and AP conducted statistical analyses and drafted the bulk of the article. AB and MP oversaw imaging protocols and the various hospitals and CG-E and NS oversaw the overall operation of the Telestroke network and CL oversaw the establishment of the network. All authors contributed to the drafting, editing, and final formation of the article on behalf of the Northern NSW Telestroke investigators.

### Conflict of Interest

The authors declare that the research was conducted in the absence of any commercial or financial relationships that could be construed as a potential conflict of interest.
