# Supplementary material for: No Evidence of the “Weekend Effect” in the Northern New South Wales Telestroke Network
Source: Front Neurol. 2020 Feb 26;11:130. doi: 10.3389/fneur.2020.00130 (PMC7057236; doi:10.3389/fneur.2020.00130)
Supplement: Supplementary file 1 [file Table_1.DOCX]

Weekend effect in an Australia telestroke network.

Supplementary tables:

| **Supplementary Table 1** | | | | | |
| --- | --- | --- | --- | --- | --- |
| Time | Hospital A  Median (IQR) | Hospital B  Median (IQR) | Hospital C  Median (IQR) | Hospital D  Median (IQR) | Hospital E  Median (IQR) |
| Door-to-needle time | 86 (67-101) | 103 (95-178) | 96 (76-130) | 80 (61-106) | 91 (72-114) |
| Door-to-call time | 38 (19-68) | 50 (25-110) | 38 (20-71) | 39 (23-60) | 48 (28-87) |
| Call-to-decision time | 40 (20-60) | 51 (30-81) | 40 (30-66) | 34 (22-50) | 38 (25-54) |
| Decision-to-needle time | 15 (10-25) | 26 (15-39) | 31 (20-87) | 27 (24-40) | 22 (19-32) |
| Door-to-imaging time | 54 (35-81) | 80 (47-126) | 55 (39-85) | 43 (21-61) | 66 (38-114) |

**Supplementary Table 1:** Median door-to-needle and component workflow times by individual hospital.

| **Supplementary Table 2** | | |
| --- | --- | --- |
| Time | β (95% CI) | P |
| Door-to-call | -7.72 (-19.99 to 4.54) | 0.217 |
| Call-to-decision | -0.65 (-6.20 to 4.90) | 0.819 |
| Decision-to-needle | -14.26 (-22.76 to -5.75) | 0.001 |
| Door-to-imaging | -1.11 (-13.62 to 11.40) | 0.861 |

**Supplementary Table 2:** Differences in work-flow times between the most experienced site (Hospital A) relative to the three least experienced sites
